# Cartilage targeting therapy with reactive oxygen species-responsive nanocarrier for osteoarthritis

**DOI:** 10.1186/s12951-022-01629-w

**Published:** 2022-09-19

**Authors:** Zengxin Jiang, Hao Wang, Zeng Zhang, Jianfeng Pan, Hengfeng Yuan

**Affiliations:** 1 Department of Orthopaedics, Shanghai Jiaotong University Affiliated Sixth People’s Hospital, Shanghai, 200233 China; 2 Institute of Microsurgery on Extremities, Shanghai Jiao Tong University Affiliated Sixth People’s Hospital, Shanghai, 200233 China; 3 Department of Orthopaedics, Zhongshan Hospital, Fudan University, Shanghai, 200032 China; 4 Department of Orthopedics, Shanghai Tenth People’s Hospital, School of Medicine, Tongji University, Shanghai, 200072 China

**Keywords:** Mesoporous silica nanoparticles, Thioketal, Reactive oxygen species, Cartilage, Nrf2

## Abstract

Targeting cartilage is a promising strategy for the treatment of osteoarthritis, and various delivery vehicles were developed to assist the therapeutic agents into cartilage. However, the underlying biomechanisms and potential bioactivities remain oversimplified. Inspired by oxidative stress in the pathogenesis of osteoarthritis, we firstly testified the antioxidant capacity of a synthetic small molecule compound, oltipraz (OL), to the chondrocytes treated by IL-1β. Then a functional reactive oxygen species (ROS) responsive nanocarrier, mesoporous silica nanoparticles (MSN) modified with methoxy polyethylene glycol-thioketal, was constructed. In vitro biomolecular results showed that compared with OL alone, MSN-OL could significantly activate Nrf2/HO-1 signaling pathway, which exhibited better ROS-scavenging proficiency and greater anti-apoptotic ability to protect mitochondrial membrane potential of chondrocytes. Further bioinformatics analysis revealed that MSN-OL suppressed clusters of genes associated with extracellular matrix organization, cell apoptosis and cellular response to oxidative stress. Animal experiments further confirmed the great cartilage-protecting ability of MSN-OL through upregulating the expression of Nrf2/HO-1 signaling pathway without obvious toxicity. In summary, this study provided a delivery system through ROS-responsive regulation of the therapeutic agents into chondrocytes of the cartilage, and confirmed the exact biological mechanisms of this innovative strategy.

**Supplementary Information:**

The online version contains supplementary material available at 10.1186/s12951-022-01629-w.

## Introduction

Osteoarthritis (OA) is a common degenerative disease of articular joints, and there are around 360 million people suffer from OA, which occurs in 50% people aged 65 years and 80% people over 80 years [1]. Non-steroidal anti-inflammatory drugs (NSAIDs) and tramadol are the most commonly administrations for the short-term relief of the symptoms, but there still lack effective interventions to prevent OA progression [2]. Therefore, the determination of new potential pharmacological targets brings expectation to the development of new therapeutic drugs for OA [3, 4].

It has been demonstrated that the level of reactive oxygen species (ROS), including hydrogen peroxides (H_2_O_2_), hydroxyl radicals (OH·), and superoxides (O^2−^), is significantly higher in the cartilage of OA patients compared with normal individuals [5, 6], while ROS-induced oxidative stress contributes to the dysfunction and apoptosis of cartilage-resident chondrocytes and extracellular matrix (ECM) degradation, which exerts a major pathogenic role in the progression of OA [7–9]. Hence, intra-articular (IA) injection of antioxidants seems an effective way to scavenge ROS and reduce the detrimental effects of ROS on chondrocytes [10–14]. Oltipraz [4-methyl-5-(2-pyrazinyl)-1,2-dithiole-3-thione, OL, structural formula shown in Additional file 1: Fig. S1] is an artificially synthesized small molecule compound that could target nuclear factor erythroid derived-2-related factor 2 (Nrf2) gene, which plays as the central for the regulation of cytoprotective and antioxidant responses [15, 16]. In addition, OL was reported in a phase II clinical trial for the treatment of nonalcoholic fatty liver disease and exhibited strong antioxidant effects [17]. However, the evidence of OL in the treatment of OA is still lacking.

The administration of small molecule compounds on OA is always by means of IA injection, which would inevitably react with the components contained in the synovial fluid and rapidly be cleared from the joint cavity. And due to many antioxidants are deficient in the ability of penetrate into the cartilage matrix, the therapeutic effects would be greatly weakened [18]. Therefore, drug-delivery systems are always required to assist the release of drugs [19–22]. In general, ideal delivery systems should not only have the excellent biocompatibility and controllable drug release ability, but could penetrate into the cartilage and get to the targeting location to perform the biological functions [23–26]. The application of mesoporous silica nanoparticles (MSN) in the field of drug delivery has been rapidly developed since Prof. Vallet-Regí and coworkers first to develop mesoporous materials to load ibuprofen as drug delivery carriers [27]. MSN have attracted much attention for their advantages on great surface area, large pore volume, easily modifiable surface, excellent biocompatibility and convenient drug-loading characteristics, which can encapsulate and control the release of a variety of biologically active molecules including small molecules, DNA, siRNA, growth factors and enzymes [28–30]. The surface of MSN could be modified with functional groups and ligands to respond to changes in the microenvironment such as abnormal temperature, acid pH, overexpressed enzymes, and excessive ROS [31–37]. In addition, thioketal (TK) was recently used as a ROS-responsive sulfur-containing linker, which has the stability to acidic and alkaline conditions but can be cleaved oxidatively [38–40]. Although TK-based ROS-responsive drug delivery carriers are not widely explored, previous report has successfully incorporated deferoxamine and TK groups into an α-cyclodextrin-based polyrotaxane platform to construct a ROS-responsive nanochelator system, which has the ability of intelligent drug release in an oxidative environment [41].

**Scheme 1:**
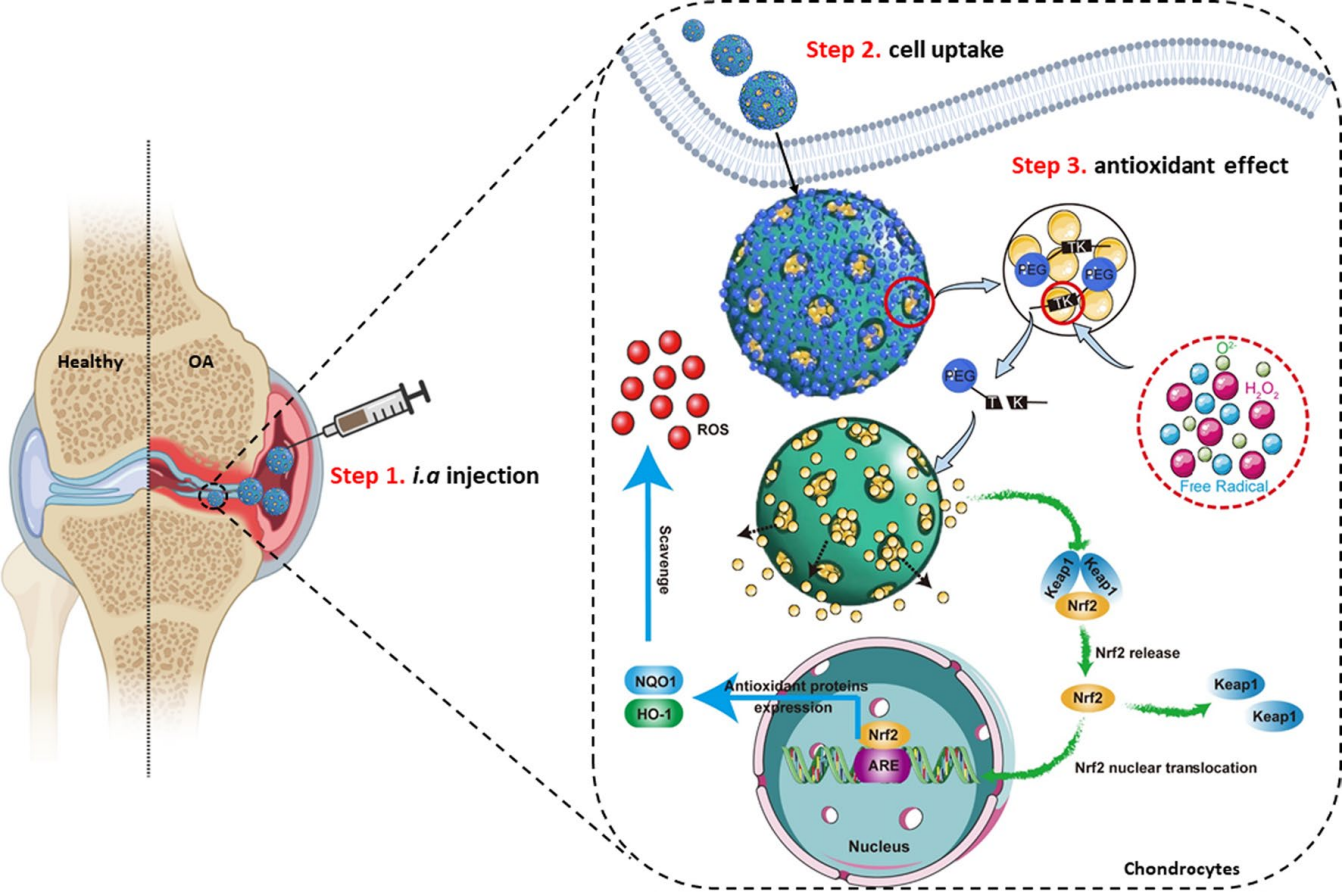
Illustration of MSN-OL for treatment of OA. Inspired by oxidative stress in the pathogenesis of osteoarthritis, a novel antioxidant, OL was loaded in the mesoporous silica nanoparticles, which was modified with methoxy polyethylene glycol-thioketal to gain the ROS-responsive ability. MSN-OL could be easily uptake by chondrocytes, and releases OL when the cells are injured and in a state of hyperoxidation. MSN-OL can further activate Nrf2/HO-1 signaling pathway and exhibit antioxidant and anti-apoptotic ability

In the present study, OL loaded MSN were modified with methoxy polyethylene glycol (mPEG) through ROS-sensitive TK groups (abbreviated as MSN-OL hereafter). The MSN formulations of OL may potentially overcome the disadvantages of simple drugs such as low solubility, instability and poor bioavailability. Moreover, the positively charged MSN could easily penetrate into cartilage and manage OL release in chondrocytes with intracellular high ROS microenvironment (Scheme 1). The underlying biomechanisms and bioactivities of MSN-OL would be elaborated as well, which is expected to provide a paradigm of drug nanocarrier study for the treatment of OA.

## Experimental section

### Materials, reagents and antibodies

MSN with cyanine-5 fluorescence (XFNANO Materials Tech. Co. Ltd., Nanjing, China); 3-mercaptopropionic acid, anhydrous acetone, N-hydroxysuccinimide (NHS), 1-ethyl-3-[3-dimethylaminopropyl]carbodiimide hydrochloride (EDC), (Sigma-Aldrich, St Louis, USA); acetonitrile, ammonium acetate and tween-80 (Aladdin Industrial Corporation, Shanghai, China); rat recombinant IL-1β (PeproTech EC, London, UK); oltipraz (Abmole Bioscience Incorporation, Shanghai, China); phalloidin-iFlour 488, primary antibodies against Nrf2, aggrecan, collagen type II and MMP13, NQO1, HO-1 (Abcam, Cambrige, UK); primary antibodies against β-Actin, Bcl-2, Bax and Caspase-3 (Abcam, Cambrige, UK); primary antibodies against Lamin B1 and MMP9 (Cell Signalling Technology Inc., Danvers, USA); horseradish-peroxidase (HRP)-labeled anti-rabbit IgG, the Annexin V-FITC/PI apoptosis detection kit, DCFH-DA, Cell Counting Kit-8 (CCK-8) and JC-1 (Beyotime Institute of Biotechnology, Shanghai, China); Dulbecco’s Modified Eagle’s Medium (DMEM), foetal bovine serum (FBS), phosphate-buffered saline (PBS), collagenase II and 0.25% trypsin (Gibco; Grand Island, NY, USA).

### Synthesis of ROS-cleavable thioketal (TK)-containing linker

A blend of 3-mercaptopropionic acid (3-MPA, 5.2 g, 49.1 mmol) and anhydrous acetone (5.8 g, 98.2 mmol) was saturated with dry hydrogen chloride. The mixture was stirred well for 4 h at room temperature. The mixture was then placed on ice to quench the reaction. After washed with hexane, the TK-containing linker was obtained.

### Synthesis and characterization of mPEG-TK

To synthesize mPEG-TK-COOH polymer, a mixture of mPEG-NH2 (1 g, 0.5 mmol), TK-containing linker (1.26 g, 5 mmol), EDC.HCl (1.15 g, 6 mmol) and NHS (0.69 g, 6 mmol) were dissolved in 20 mL of DCM and stirred well for 4 h at room temperature. Rotating evaporation was used to remove the solvent and cold ether was added to precipitate the product. After vacuum drying, the product was dissolved in DMF and dialyzed against distilled water using an MWCO: 3500 Da dialysis membrane for three days. After freeze-drying, the expected mPEG-TK-COOH was collected as a white powder. After that, the ^1^H-Nuclear magnetic resonance (NMR) spectra were obtained on a VNMRS-400 (VARIAN, USA) spectrometer at 400 MHz by using CDCl_3_ as the solvent with TMS as an internal standard.

### Preparation of MSN-OL

15 mg MSN and 5 mg oltipraz were fully dissolved in 166μL DMSO by the aid of sonication and incubated overnight at 37 °C to promote OL completely adsorbed into MSN. The mixture was centrifuged at 18,000 rpm for 20 min to separate the supernatant and the sediment. The supernatant is retained for the subsequent synthesis process and measurement of drug loading and encapsulation efficiency. 1.5 mg mPEG-TK was dissolved in 10 μL supernatant and then added it to the previous precipitate with 0.3 mg EDC for 3 h reaction. The nanoparticles were separated by centrifugation at 18,000 rpm and washed with pure water three times, re-dispersed in ultrapure water at a concentration of 10 mg/mL and stored in the dark at 4 °C for subsequent study.

Drug loading efficiency (LE) and encapsulation efficiency (EE%) determination: The OL in the supernatant mentioned above were quantified using high-performance liquid chromatography (HPLC, Shimadzu, Japan) according to the pre-established standard curve. The LE and EE% were calculated according to the following formulas:

LE = weight of encapsulated OL/weight of OL-loaded nanoparticles.

EE% = weight of encapsulated OL/weight of the total OL × 100%

### Characterization of nanoparticles

The nanoparticles were observed by transmission electron microscopy (TEM, JEM-200CX, JEOL, Akishima, Tokyo, Japan) and scanning electron microscopy (SEM, ULTRA plus; Zeiss, Zurich, Switzerland). Dynamic light scattering (DLS) was performed to determine particle size and zeta potential on a Zetasizer Nano ZS90 (Malvern, Worcestershire, UK). Nanoparticles were observed under a confocal microscopy (FV3000, Olympus, Tokyo, Japan).

### Cell isolation and culture

All animal experiments were approved by the Ethics Committee on Animal Experiments of Shanghai Sixth People’s Hospital (No. 2021-0194). Six 4-week-old male Sprague Dawley (SD) rats (Shanghai SLAC laboratory animal CO.LTD) were euthanized using CO_2_ inhalation. Death was confirmed by cardiac and respiratory arrest. The cartilages were harvested from the rat joints and minced into small pieces. Cartilages were digested with a 0.25% trypsin solution for 30 min and subsequently digested with 0.1% collagenase II solution for 12 h. The chondrocytes were collected and cultured in Dulbecco’s Modified Eagle’s Medium (DMEM) supplemented with 10% foetal bovine serum (FBS) at 37 °C and 5% CO_2_.

### Nanoparticles cellular uptake determination in vitro

After treatment for 24 h, chondrocytes were washed with PBS and then fixed with 4% paraformaldehyde for 20 min at room temperature. Chondrocytes were stained with Alexa Flour 488-conjugated phalloidin (1:500) for 45 min followed with hoechst for 5 min. Chondrocytes were observed under confocal microscopy (magnification, × 200). Additionally, chondrocytes were collected and washed with PBS 3 times to remove the supernatant. Chondrocytes were re-suspended in 500 μL of PBS and analyzed using flow cytometry to quantify the cellular uptake of the nanoparticles at the APC channel.

### Quantification of released OL

1 mL of MSN-OL suspension was transferred to a dialysis tube, and the dialysis tube was immersed in 10 mL of PBS with or without 0.1% hydrogen peroxid. The tube was stirred at 100 rpm at 37 °C. At predetermined time intervals (6, 12, 24, 48 and 72 h), the release medium was collected and the fresh PBS was then added. Concentrations in the release medium were determined by UV–visible spectra with a UV–visible spectrophotometer (FlexStation 3, Molecular Devices, United States).

### Cell viability assay

Chondrocyte were seeded in 96-well plates at a dense of 10,000 cells/well. Cells were treated with different concentrations of MSN-OL (30, 60, 120, 240, 480 μg/mL) for 24 h and 48 h. Chondrocytes were incubated with serum-free DMEM containing 10% Cell Counting Kit-8 (CCK-8) solution at 37 °C for 2 h. The absorption was evaluated at 450 nm using a spectrophotometer (FlexStation 3, Molecular Devices, United States).

### ROS levels evaluation

ROS levels in chondrocytes were evaluated using the ROS sensitive dye 2′,7′-dichlorofluorescein diacetate (DCFH-DA). Briefly, chondrocytes were stained with 10 μM DCF-DA for 30 min and then washed with serum-free DMEM three time to remove the residual extracellular DCFH-DA. Flow cytometry analysis were conducted using a BD Accuri C6 plus flow cytometer (BD Biosciences, Vianen, The Netherlands).

### Mitochondrial membrane potential determination

The mitochondrial membrane potential of chondrocytes was assessed using JC-1 staining. Chondrocytes were collected and stained with JC-1 (5 µg/ml) for 25 min at 37 °C. Cells were washed with PBS 3 times again to remove the residual JC-1. Flow cytometry analysis were conducted using a BD Accuri C6 plus flow cytometer.

### Cell apoptosis evaluation

Apoptosis analysis was conducted Annexin V-fluorescein isothiocyanate (FITC)/propidium iodide (PI) staining. Chondrocytes were collected and stained with 300 μl binding buffer containing 5 μL Annexin V followed with 200 μL binding buffer containing 5 μL PI for 5 min at 37 ℃ in the dark. Flow cytometry analysis were conducted using a BD Accuri C6 plus flow cytometer within 30 min.

### Western blotting

Western blotting was performed to evaluate protein expression. Total protein was extracted using radioimmunoprecipitation assay buffer while nuclear protein was extracted using a nuclear protein extraction kit. Western blot analysis was performed as previously described. The blots were visualized using enhanced chemiluminescence on an imaging system (Tanon, Shanghai, China) while integrated fluorescence intensity was analyzed using ImageJ software (version 1.8.0; National Institutes of Health, USA).

### Bioinformatics analysis

Cartilages from the joints of rats were used for ex-vivo cartilage explants culture. Ex-vivo cartilage explants were divided into four groups: control group, IL-1β group, IL-1β + OL group and IL-1β + MSN-OL group. The concentrations of IL-1β, OL and MSN-OL were 10 ng/mL, 40 µM and 0.27 mg/mL, respectively. After 3 days, total RNA was extracted and the transcriptome was sequenced o using the Illumina sequencing platform. Differentially expressed genes (DEGs) were analyzed with the DESeq2 package (1.34.0). Gene ontologies were analyzed to identify different biological process.

### Rat OA model

In vivo study was performed using 90 eight-week-old SD rats with six rats in each group. Anterior cruciate ligament transection (ACLT) was performed to establish rat osteoarthritis model as previously described [42]. The right knee joint is the side of the operation. Rats were randomized to sham group, ACLT group, ACLT + OL group, ACLT + MSN-NC group and ACLT + MSN-OL group. Each group had three time points of 4, 8 and 12 weeks.

### Nanoparticles absorption and degradation in vivo

OA rats received an IA injection of 100 μL nanoparticles (7 mg/mL). Fluorescence images were taken using the In Vivo Imaging System (IVIS) Spectrum (Perkin Elmer, Santa Clara, CA) and IVIS imaging software (Perkin Elmer, Santa Clara, CA) at day 0 (after IA injection immediately), day3, day7, day14 and day21. The cartilages were used to make frozen sections. The sections were stained with DAPI and scanned using an Olympus BX51 microscope (Olympus, Tokyo, Japan).

### Behavioral evaluation (gait analysis)

Gait analysis was performed using the Catwalk automated gait analysis system (Noldus Information Technology, The Netherlands). Each rat walked freely on a glass lit by green light. Paw prints were then digitized. The whole process was recorded by the video camera. Data was collected and analyzed using catwalk program software (Noldus, CatWalk XT version 10.6.608).

### Radiographic analysis

Micro-computed tomography (microCT) scans of the joints were preformed using SCANCO 50 (Switzerland) at week 12. Three-dimensional reconstructed images of the knee joints were generated. The BV/TV were analyzed.

### Histopathology analysis

Organs including heart, liver, kidney and knee joint were harvested and fixed in 10% formalin at week 4, week 8 and week 12. As to the knee joints, after decalcified in 10% ethylenediamine tetraacetic acid (EDTA) for 4 weeks, tissues were dehydrated, embedded in paraffin and cut into 5 μm thick slices with an ultrathin semiautomatic microtome (RM2016, Leica, Germany). The sections were stained with H&E, Safranin O/Fast Green and toluidine blue. Immunohistochemistry (IHC) of collagen type II, HO-1 and NQO1 were also performed. The sections were scanned using a microscope. OARSI score, Mankin score and expression levels of collagen type II, HO-1 and NQO1 were evaluated [43, 44].

### Statistical analysis

Each experiment was independently performed three times. Data are presented as the mean ± standard deviation. Students *t-test* was used to compare the two groups. Statistical comparisons among multiple groups were performed using one-way analysis of variance (ANOVA) followed by Tukey multiple comparison test or a Mann–Whitney test (nonparametric, OARSI score and Mankin score). P values < 0.05 were considered statistically significant. All statistical analyses were performed using SPSS software (version 22.0; IBM Corp.).

## Results and discussion

### Anti-oxidative effects of OL on chondrocytes

The cytotoxic effects of different concentrations of OL (0, 0.625, 1.25, 2.5, 5, 10, 20, 40, 80 and 160 µM) on chondrocytes were firstly evaluated by Cell Counting Kit-8 (CCK-8) assay at 24 and 48 h. OL were not toxic to chondrocytes even at concentrations as high as 160 μM at 24 and 48 h (Additional file 1: Fig. S2). We further explored the antioxidative effects of OL in chondrocytes. We found both 20 and 40 µM OL could effectively scavenge excessive ROS induced by 10 ng/mL IL-1β in chondrocytes (Additional file 1: Fig. S3). IL-1β was often used to simulate cartilage inflammation and mimic the inflammatory microenvironment in chondrocytes [45], while 20 and 40 µM OL increased Nrf2 protein level in the nucleus and enhanced HO-1 and NQO1 protein expression in chondrocytes exposure to IL-1β, suggesting OL could significantly activate Nrf2/HO-1 signaling pathway to exert antioxidative effects (Additional file 1: Fig. S4). In addition, 20 and 40 µM OL significantly enhanced the protein expression of collagen type II and aggrecan while inhibited the protein expression of MMP9 and MMP13 in chondrocytes exposure to IL-1β, indicating OL could prevent ECM degradation in vitro (Additional file 1: Fig. S5). Moreover, OL treatment without IL-1β intervention didn’t change the expression of collagen II, aggrecan, MMP9 and MMP13, although it increased the expression of nrf2 pathway-related proteins. What’s more, Safranin O and toluidine blue staining of ex vivo cultured experiment cartilage explants showed that the glycosaminoglycan (GAG) gradation was prevented by OL, further verified the ability of OL to protect cartilages from ECM gradation (Additional file 1: Fig. S6).

Although the results above confirmed the effectiveness of OL in preventing chondrocyte oxidative damage and cartilage degeneration in vitro, the therapeutic efficacy of OL might not be as high as expected in vivo due to the fast clearance and poor penetrative ability. The subsequent study also proved the low therapeutic efficacy of OL in OA rat model as described later. Therefore, we constructed a customized carrier for OL.

### Preparation and physical properties of MSN-OL

MSN were used as OL carrier for their advantages on great surface area, large pore volume, easily modifiable surface, excellent biocompatibility and convenient drug-loading properties [28, 29]. Carrying Cy5 fluorescence on the nanoparticles facilitated to trace the nanoparticles in vitro and in vivo experiments. TK is one of the most used chalcogen-containing responsive linkers [39]. TK-modified drug delivery systems showed great ability to manage drug release in an oxidative environment [40]. To ensure that OL was preserved in MSN for as long as possible before ROS stimulation, the mPEG-TK was synthesized to enable the MSN to obtain the ROS-responsive properties. Fluorescence spectra of MSN and MSN-OL at 630 nm were shown in Additional file 1: Fig. S7. The results showed that maximum emission wavelength of MSN and MSN-OL were 685 nm and 693 nm, respectively. OL loading leaded to a shift of about 8 nm in maximum emission wavelength. These changes were acceptable because they did not hinder the nanoparticles localization fluorescently.

**Fig. 1:**
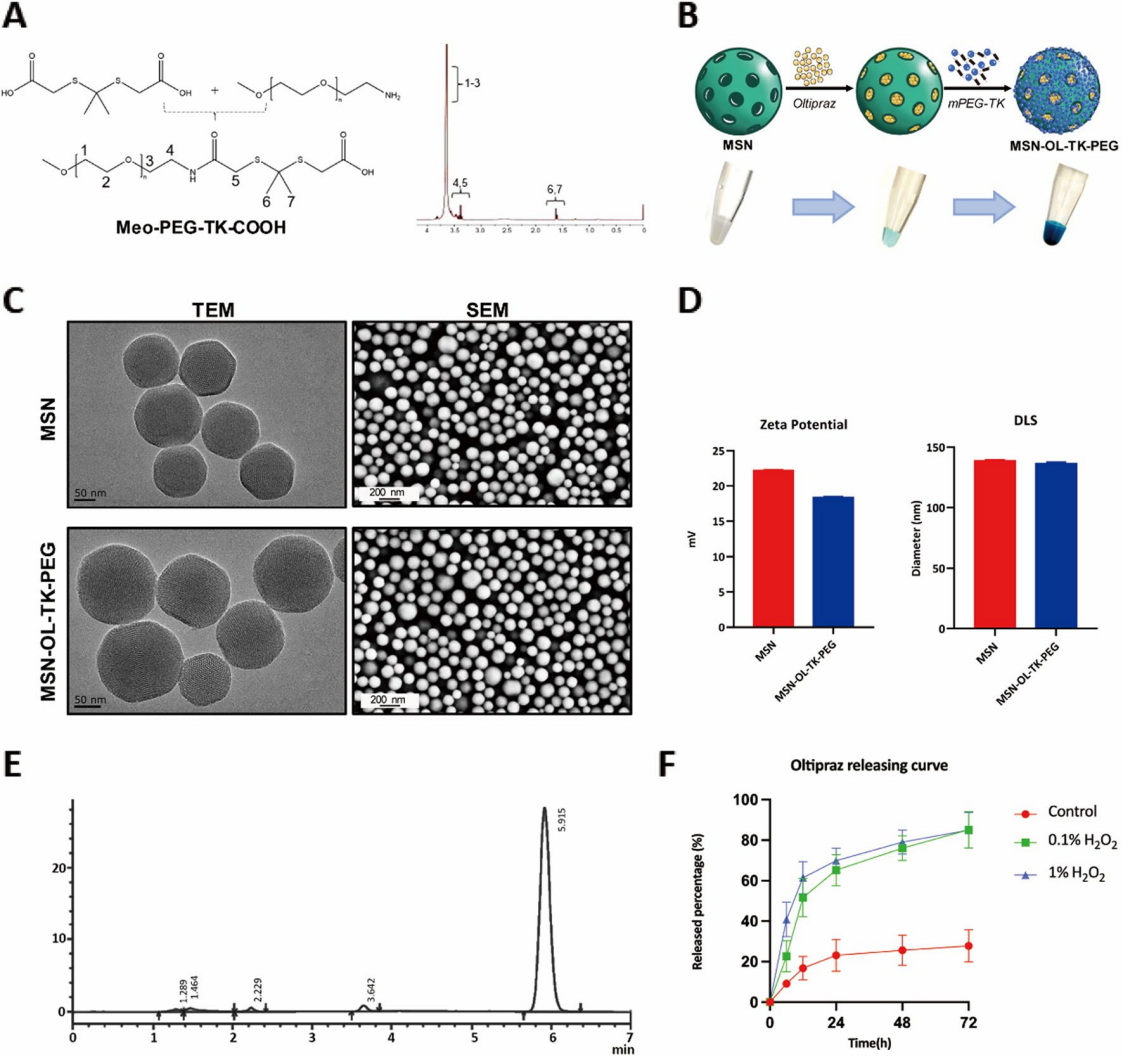
Preparation and characterization of MSN-OL. **A** Synthesis and ^1^H-NMR characterization of mPEG-TK. **B** Fabrication process and digital photos of the MSN-OL in the reaction process. **C** TEM and SEM were used to observe the morphology of the nanoparticles. **D** The zeta potential and dynamic light scattering characteristics of MSN-OL. **E** HPLC results of drug loading rate and encapsulation rate of MSN-OL. **F** OL releasing curve was evaluated with or without the treatment of hydrogen peroxide

The synthesis of the mPEG-TK- was shown in Fig. 1A, and the ^1^H-NMR spectrum result demonstrated the successful synthesis process. The chemical shifts of protons on the polymer were identified by the numbers. Next, the route of MSN-OL synthesis was displayed in Fig. 1B. MSN in the aqueous solution displayed white colour, MSN-NC displayed light blue colour while MSN-OL dark blue colour. TEM and SEM imaging showed that both the bare MSN and MSN-OLwere well-distributed with uniform size and spherical shape (Fig. 1C). The results of zeta potential assay showed that MSN and MSN-OL were positively charged with a zeta potential value of about 22 mV and 18 mV, respectively (Fig. 1D, Additional file 1: Fig. S8), suggesting that the surface carried a strong positive charge. The results of DLS showed that the MSN and MSN-OL had average diameters of 138 nm and 135 nm, respectively, suggesting that the average sizes of nanoparticles did not change obviously after modified by TK-mPEG with OL loading (Fig. 1E, Additional file 1: Fig. S9). Results based on the analysis of high-performance liquid chromatography (HPLC) showed that drug loading efficiency (LE) was about 3.55% and encapsulation efficiency (EE%) was approximately 11.05% (Fig. 1F). The in vitro OL release characteristics of MSN-OL were then evaluated. The OL release rate of MSN-OL was 9.1%, 16.8%, 23.1%, 25.6%, 27.8% at 6, 12, 24, 48 and 72 h respectively when incubated in PBS, while the release rate improved to 22.7%, 51.6%, 65.2%, 76.1%, 85.2% at 6, 12, 24, 48 and 72 h when PBS containing 0.1% H_2_O_2_, respectively, and the release rate further improved to 40.9%, 61.4%, 69.9%, 79.1%, 84.9% at 6, 12, 24, 48 and 72 h when PBS containing 1% H_2_O_2_ (Fig. 1F). Initial burst release of OL was observed within 12 h followed by sustained release with cumulative drug release of about 80% at 72 h in PBS containing 0.1% H_2_O_2_. The release rate of OL did not show a significant relative drug-release burst in PBS, suggesting redox environment promoted the OL release from MSN-OL.

### Enhanced anti-oxidative and anti-apoptosis effects of MSN-OL on chondrocytes

**Fig. 2:**
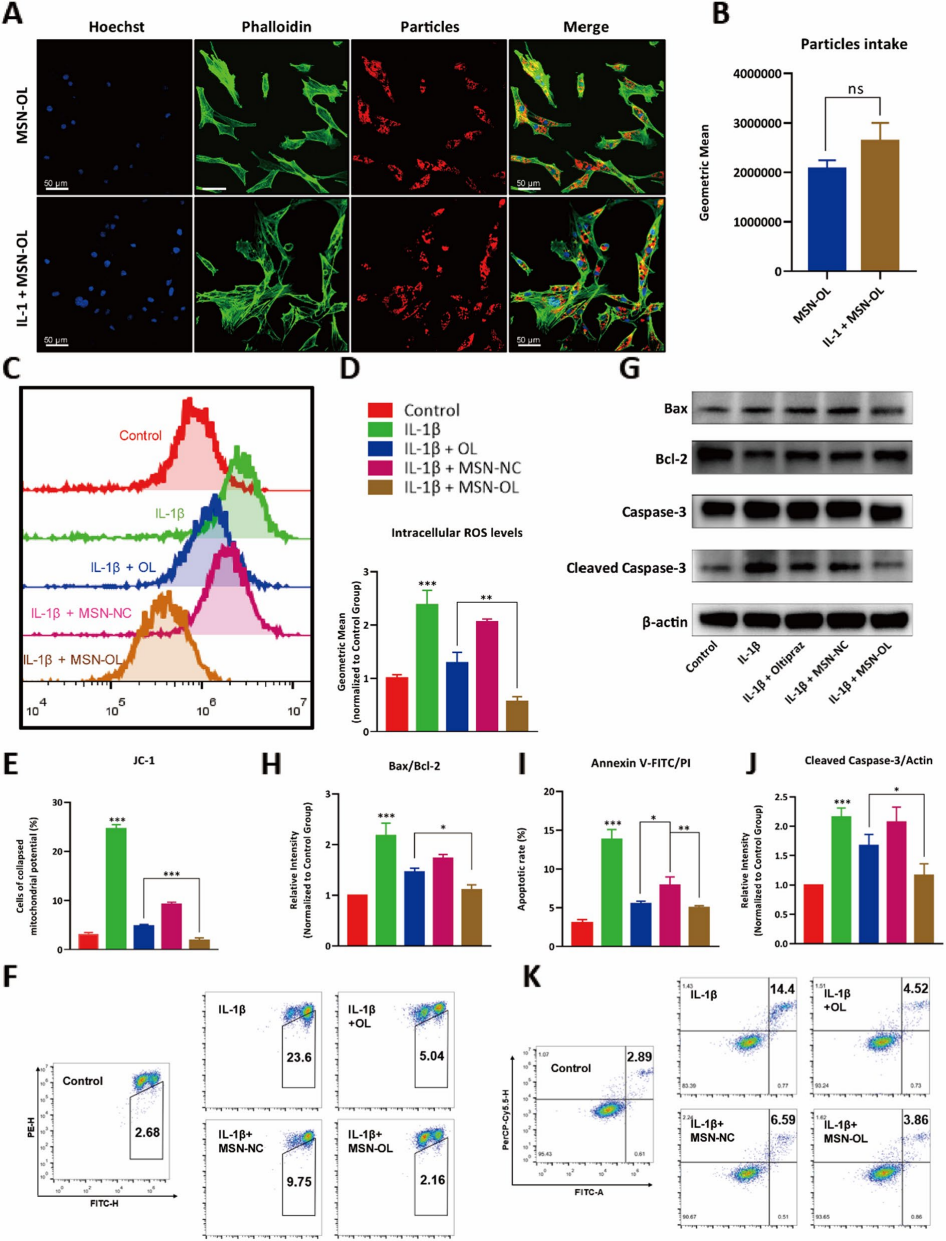
Antioxidant and anti-apoptotic effects of MSN-OL in vitro. **A**, **B** The laser scanning confocal microscope and flow cytometry analysis were used to determine the uptake of nanoparticles. **C**, **D** Intracellular ROS levels were measured by DCFH-DA and flow cytometry. **E**, **F** Mitochondrial membrane potentials of chondrocytes were determined with JC-1 staining and flow cytometry. **K**, **I** The apoptotic rate of chondrocytes was determined using Annexin V-FITC/PI staining. **G**, **H**, **J** Mitochondrial apoptotic pathway-related proteins, such as bax, bcl-2 and cleaved caspase-3, were measured using Western blotting. (* indicated p < 0.05, ** indicated p < 0.01, *** indicated p < 0.001 in comparison with the IL-1β or IL-1β + OL treatment group, respectively)

As the nanoparticles would be injected into the joint cavity directly, the in vitro cytotoxicity of MSN-OL on chondrocytes was firstly determined. MSN-OL concentrations as high as 480 μg/mL were tested and even in this case no significant cell toxicity was observed (Additional file 1: Fig. S10). Assuming that all the OL loaded in 480 μg/mL MSN-OL was completely released, the concentration of OL was only about 70.56 μM according to EE%, which was still non-toxic. Therefore, MSN-OL concentrations equal or less than 480 μg/mL were safe for chondrocytes, suggesting an excellent biocompatibility. We then assessed the uptake of nanoparticles in vitro. Chondrocytes were stained with phalloidin and hoechst and then observed under a confocal microscopy. Nanoparticles were clearly inside the cells, demonstrating the ability of chondrocytes to actively uptake nanoparticles with or without the existence of IL-1β (Fig. 2A). Flow cytometry analysis was also used to quantify the cellular uptake of the nanoparticles (Additional file 1: Fig. S11). The results showed the IL-1 treated group cells took up more nanoparticles than control cells did, though this difference was not statistically significant (Fig. 2B). We further explored the antioxidative effects of MSN-OL in vitro, in which 40 μM OL was used in OL treatment group and 0.27 mg/mL in MSN-OL group, which was equal to the OL concentration in the OL treatment group after calculation. The results of the DCF staining showed that MSN-OL more potently inhibited the production of ROS compared with OL treatment (Fig. 2C, D).

In addition, the apoptosis of chondrocytes was evaluated. The decrease of mitochondrial membrane potential is a hallmark of early apoptotic events [46], and the results of JC-1 staining showed that the MSN-OL was better able to alleviate the mitochondrial damages in chondrocytes than the OL (Fig. 2E, F). Moreover, the annexin V-FITC/PI staining results also indicated that MSN-OL treatment could reduce the apoptotic chondrocyte rate compared with OL (Fig. 2K, I). Further western blotting confirmed that both MSN-OL and OL could reduce the expression levels of Bax and cleaved caspase-3, and improve the expression of Bcl-2 in chondrocytes treated with IL-1β. (Fig. 2G) MSN-OL reduced the Bax/Bcl-2 ratio and cleaved caspase-3 level compared with OL, suggesting that MSN-OL had a stronger ability to prevent IL-1β induced chondrocyte apoptosis (Fig. 2H, J). Overall, compared with OL treatment, MSN-OL was able to exert better cytoprotection from inflammation-induced early and late apoptosis.

### Up-regulation of Nrf2-HO-1 signaling by MSN-OL

**Fig. 3:**
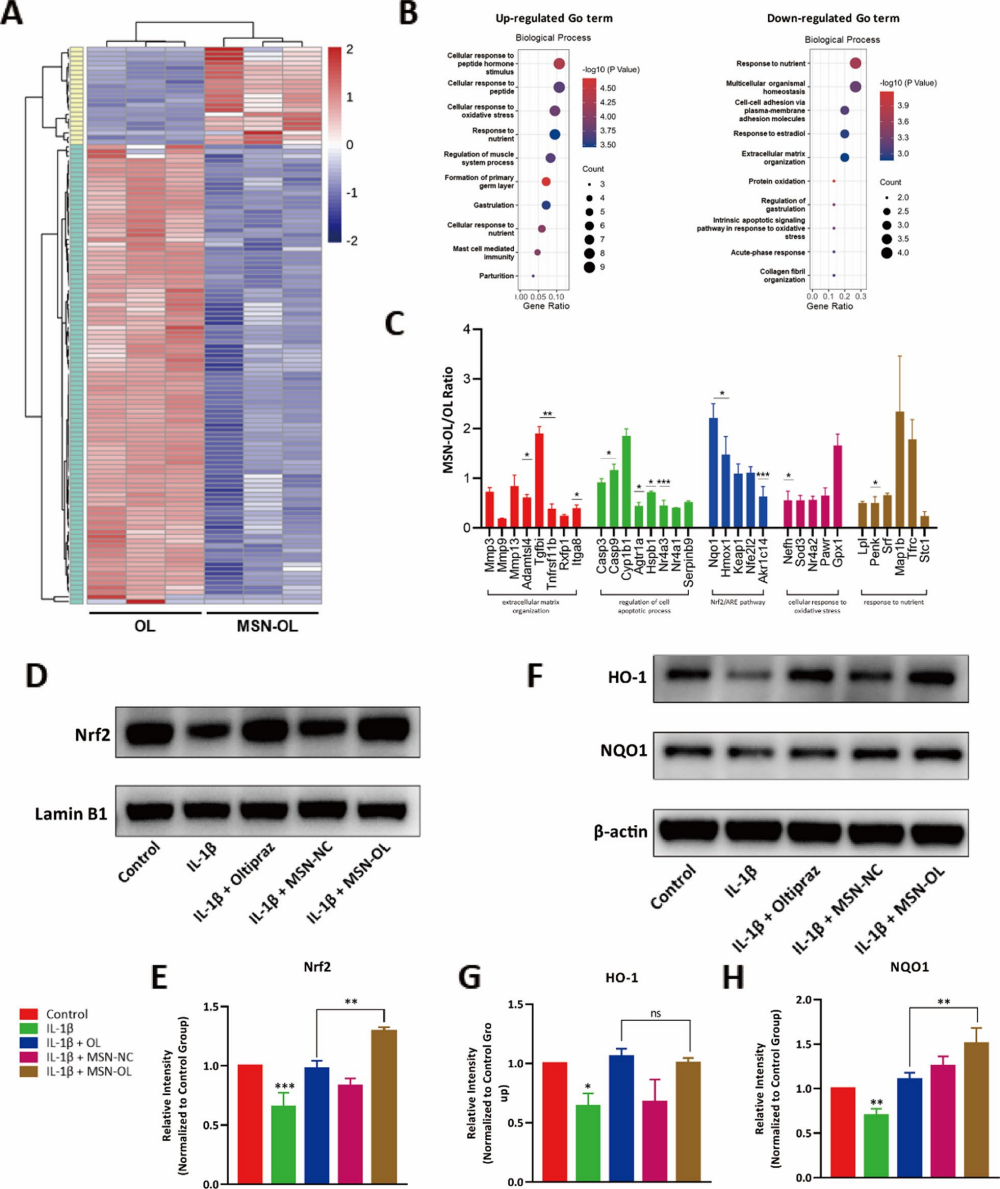
Up-regulation of Nrf2-HO-1 signaling by MSN-OL. **A** Clustering analysis of the differentially expressed genes (DEGs) in cartilages treated by OL or MSN-OL. **B** Gene Ontology analysis was performed to clarify the differences in biological process. **C** Five terms of interest with DEGs were analyzed. **D**–**H** Western blot was used to determine the changes of Nrf2/HO-1 signaling pathway between different groups, respectively. (* indicated p < 0.05, ** indicated p < 0.01, *** indicated p < 0.001 in comparison with the OL in (**C**), and IL-1β or IL-1β + OL treatment group in the rest figures, respectively)

To further confirm that MSN-OL enhanced the biofunctions of OL, we established ex-vivo cultured cartilage explants model. Illumina transcriptome sequencing technology was used to sequence the transcriptomes of cartilage explants between IL-1β + OL group and IL-1β + MSN-OL group. Differentially expressed genes (DEGs) were identified and Gene Ontology (GO) analysis was performed to clarify the differences in biological process (Fig. 3A, B). Five terms of interest with DEGs, including ECM organization, regulation of cell apoptotic process, Nrf2/ARE pathway, cellular response to oxidative stress and response to nutrient, were shown in Fig. 3C, in which the results confirmed that MSN-OL enhanced the biofunctions of OL and presented as a stronger activation of Nrf2 signaling pathway. Moreover, western blotting analysis further confirmed that compared with OL treatment, MSN-OL increased Nrf2 protein level in the nucleus (Fig. 3D, E) and enhanced NQO1 protein expression in chondrocytes under IL-1β treatment (Fig. 3F–H). Therefore, we believed that MSN-OL enhanced the biofunctions of OL and thus exerted better effects on preventing oxidative impairment.

### In vivo drugs release behaviours and biotoxicity

**Fig. 4:**
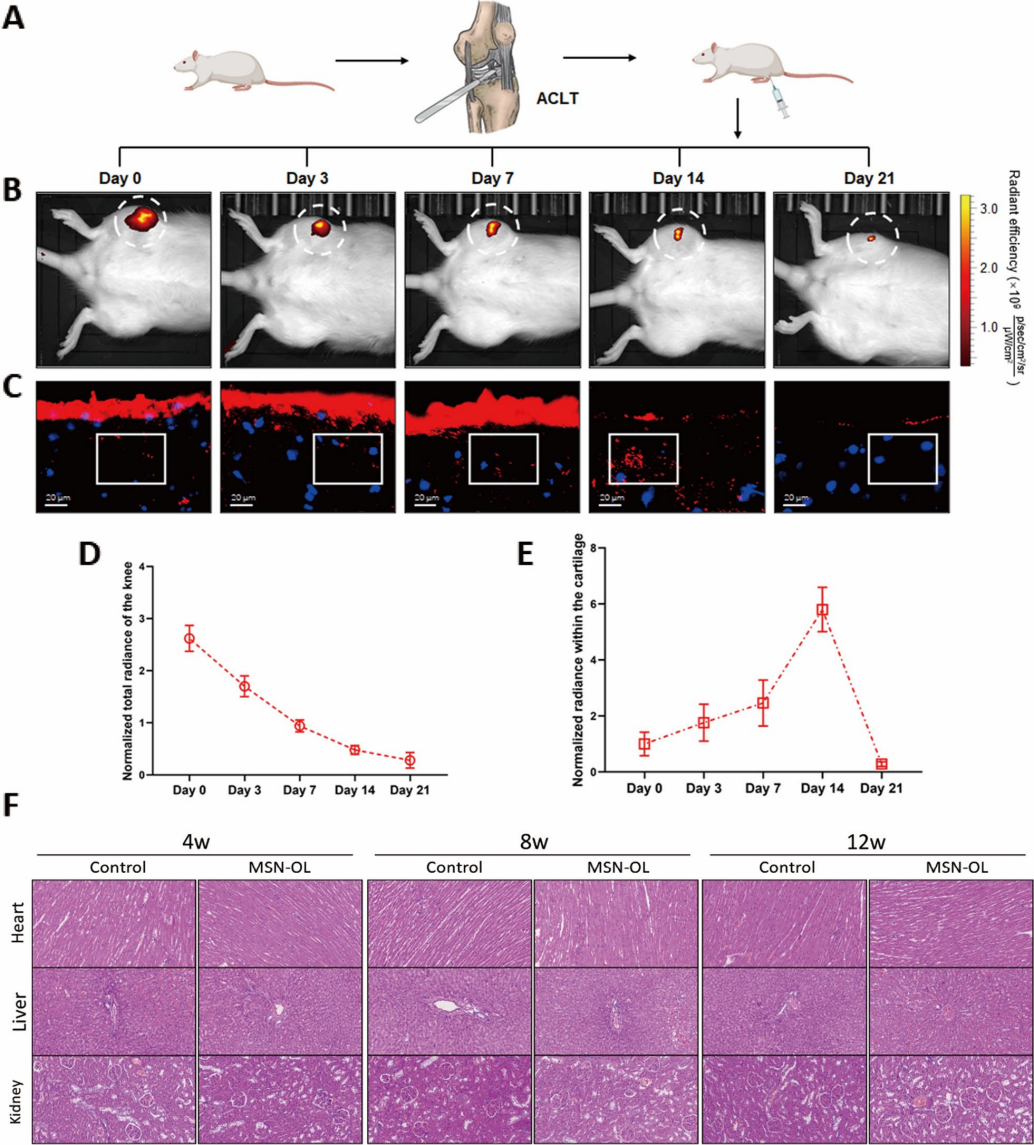
In vivo drugs release behaviours and biotoxicity. **A** The schematic of single intraarticularly injection test. **B** Fluorescence imaging were used to observe the MSN-OL degradation in the knee joint cavity. **C** Frozen slicing technology were used to trace the nanoparticle degradation within the cartilage. **D**, **E** Analysis results showed that MSN-OL degraded with time in the joint cavity but reached the peak within the cartilage after 2 weeks. **F** Representative images of heart, liver, and kidney H&E staining at 4 weeks, 8 weeks, and 12 weeks

To investigate whether MSN-OL could take drugs into cartilage, Perkin Elmer in-vivo imaging system was used to observe the fluorescence signal in rats at day 0 (after a single IA injection immediately), day 3, day 7, day 14 and day 21 (Fig. 4A, B). The fluorescence intensity allowed a semiquantitative estimation of MSN-OL degradation. A decline of fluorescence intensity over time indicated gradual degradation and clearance of MSN-OL (Fig. 4D). The fluorescence in the knee joint can be observed within 21 days after joint injection using live imaging, indicating MSN-OL could stay in the joint for at least 21 days.

Additionally, frozen cartilage sections were prepared at various time points after IA injection. Slicers were stained with hoechst and observed under fluorescence microscope. Fluorescence images showed that a large number of MSN-OL particles adhered to the cartilage surface and gradually entered cartilage matrix and cells after injection (Fig. 4C). Analysis results indicated that MSN-OL degradation reached the peak after 2 weeks. At day 21, the MSN-OL on the cartilage surface were almost disappeared (Fig. 4E). These findings showed that MSN-OL as a drug carrier could effectively take drugs into cartilage and target cells, and thus overcome the shortcomings of being cleared away quickly from the joint cavity.

Histological analysis of the major organs (heart, liver and kidney) of rats that stained with hematoxylin and eosin (H&E) was performed at 4, 8 and 12 weeks to evaluate the potential toxicity of MSN-OL. No obvious pathological changes such as degeneration and necrosis were observed, indicating MSN-OL did not lead to obvious organ toxicity (Fig. 4F).

### Therapeutic effects and mechanisms of MSN-OL on rat OA model

Anterior cruciate ligament transection (ACLT) was performed to establish the animal OA model according to the previous studies [42, 47]. Knee surgery-induced joint destabilization would result in degradation of articular cartilage and changes of subchondral bone. After calculation, the dose of OL in the OL group (100 μM, 100 μL) were consistent with the dose in the MSN-OL group (7 mg/mL, 100 μL) in a single IA injection. The injections were performed every two weeks and the rats were scarified after behavioural evaluation at 4 weeks, 8 weeks and 12 weeks.

**Fig. 5:**
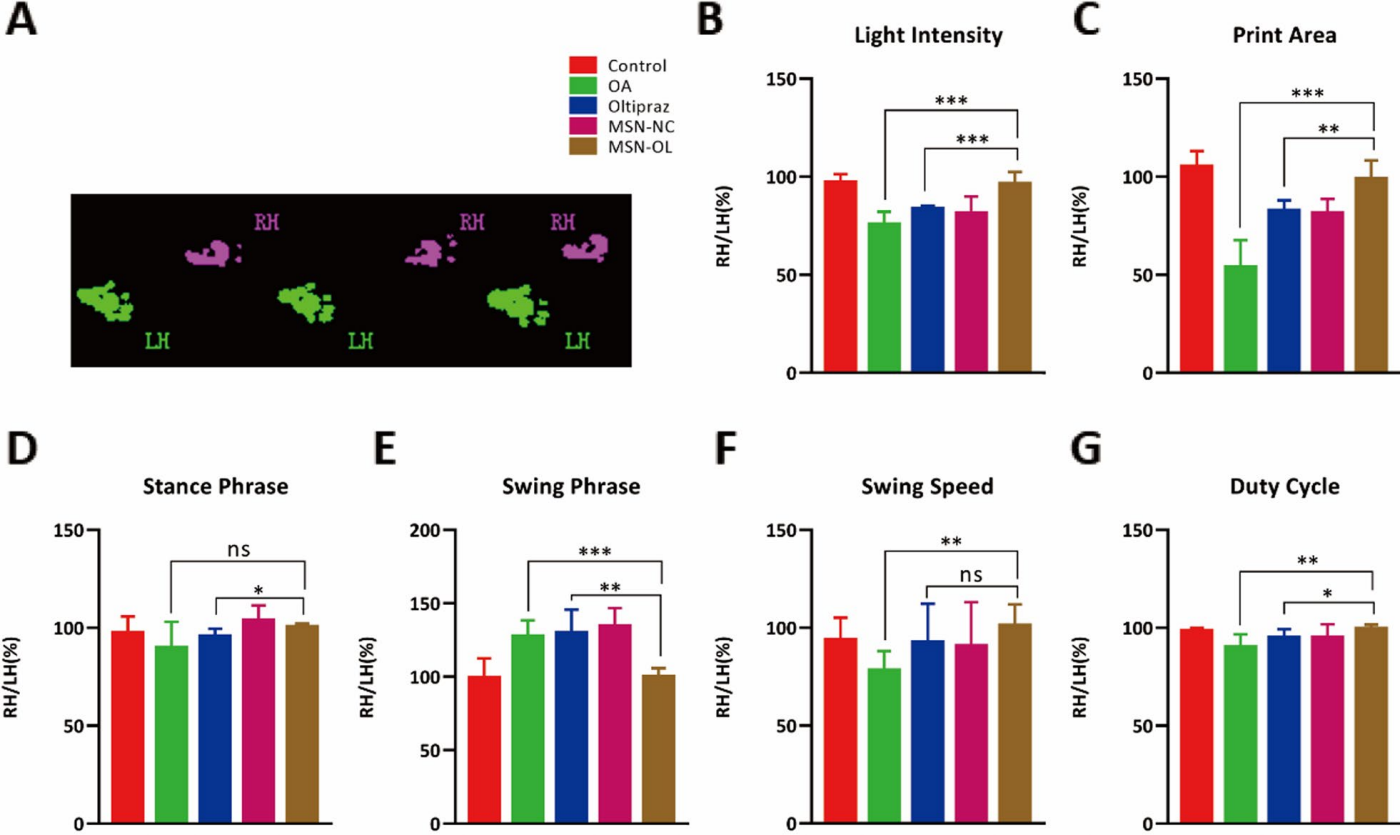
Changes of animal behavior after MSN-OL treatment. **A** Gaits of rats were analyzed using Catwalk gait analysis system. **B**–**G** Indicators including light intensity, print area, duty cycle, swing phrase, stance phrase, swing speed were compared between different groups. (* indicated p < 0.05, ** indicated p < 0.01, *** indicated p < 0.001 in comparison with OA or the OL treatment group, respectively)

**Fig. 6:**
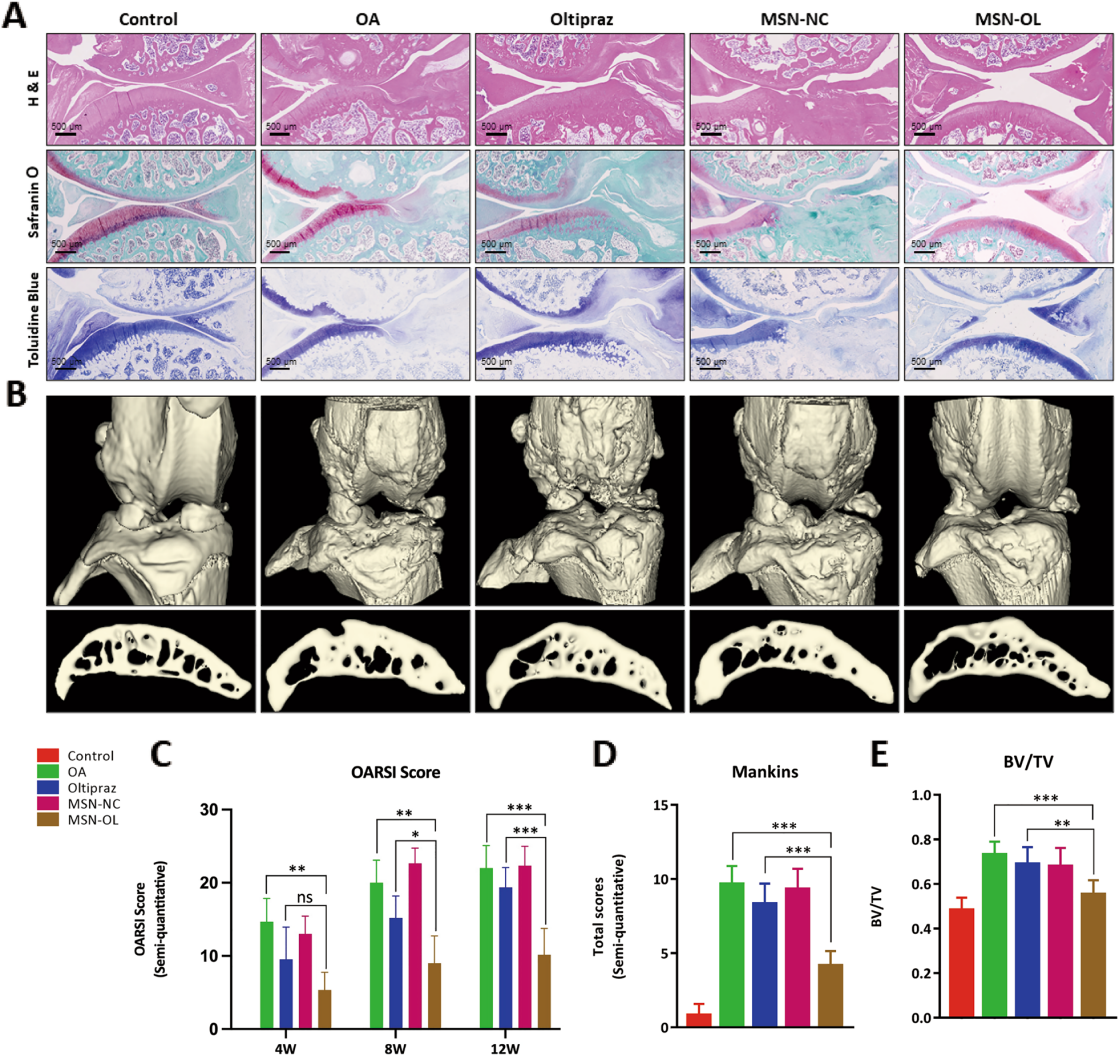
MSN-OL alleviated the disease progression in rat OA model. **A** Representative images of H&E, safranin O/fast green and toluidine blue staining for each group at 12 weeks. **B** MicroCT 3D renderings of knee joints and subchondral bone at 12 weeks. **C**, **D** OARSI scores and total Mankin scores of articular cartilage. **E** The BV/TV analysis results of different groups. (* indicated p < 0.05, ** indicated p < 0.01, *** indicated p < 0.001 in comparison with OA or the OL treatment group, respectively)

The results of gait analysis (Fig. 5A) showed that MSN-OL improved light intensity, print area, duty cycle and swing phrase while OL only improved the print area in OA rats at 12 weeks (Fig. 5B, C, E, G). There was no statistical difference in stance phrase (Fig. 5D) between OA and MSN-OL groups, and swing speed (Fig. 5F) between OL and MSN-OL groups. In addition, H&E and Safranine O-Fast Green staining showed that the surface of the condylar cartilages in the control group were intact and smooth at 4, 8 and 12 weeks (Fig. 6A, Additional file 1: Fig. S12, S13). Rough cartilage surface of cartilage, abnormal subchondral bone remodeling and reduced glycosaminoglycan in the cartilage were observed in OA group. At 4 weeks, OL treatment showed a certain cartilage protection effect. Although cartilage was worn and glycosaminoglycans reduced, there was no obvious contour change. Moreover, no obvious changes were found in the cartilage surface, histological structure of subchondral bone and the content of glycosaminoglycans in MSN-OL group. At 8 and 12 weeks, the OL group experienced severe cartilage wear, degradation of cartilage matrix, and subchondral bone remodeling. In contrast, the cartilage was still almost intact in the MSN-OL group even at 12 weeks. OARSI grades (Fig. 6C) and Mankin score (Fig. 6D) were further used to assess the severity of articular cartilage degradation. Samples in MSN-OL group had lower OARSI scores and Mankin scores, suggesting MSN-OL showed better therapeutic effects on OA. The joints were then scanned using micro-CT for further analysis of the subchondral bone, and the percent bone volume (BV/TV) was included. Significant increase of BV/TV was observed in OA and OL treatment groups, while MSN-OL significantly reduced the BV/TV, suggesting that MSN-OL could protect subchondral bone from sclerosis in OA rats as well (Fig. 6B, E).

**Fig. 7:**
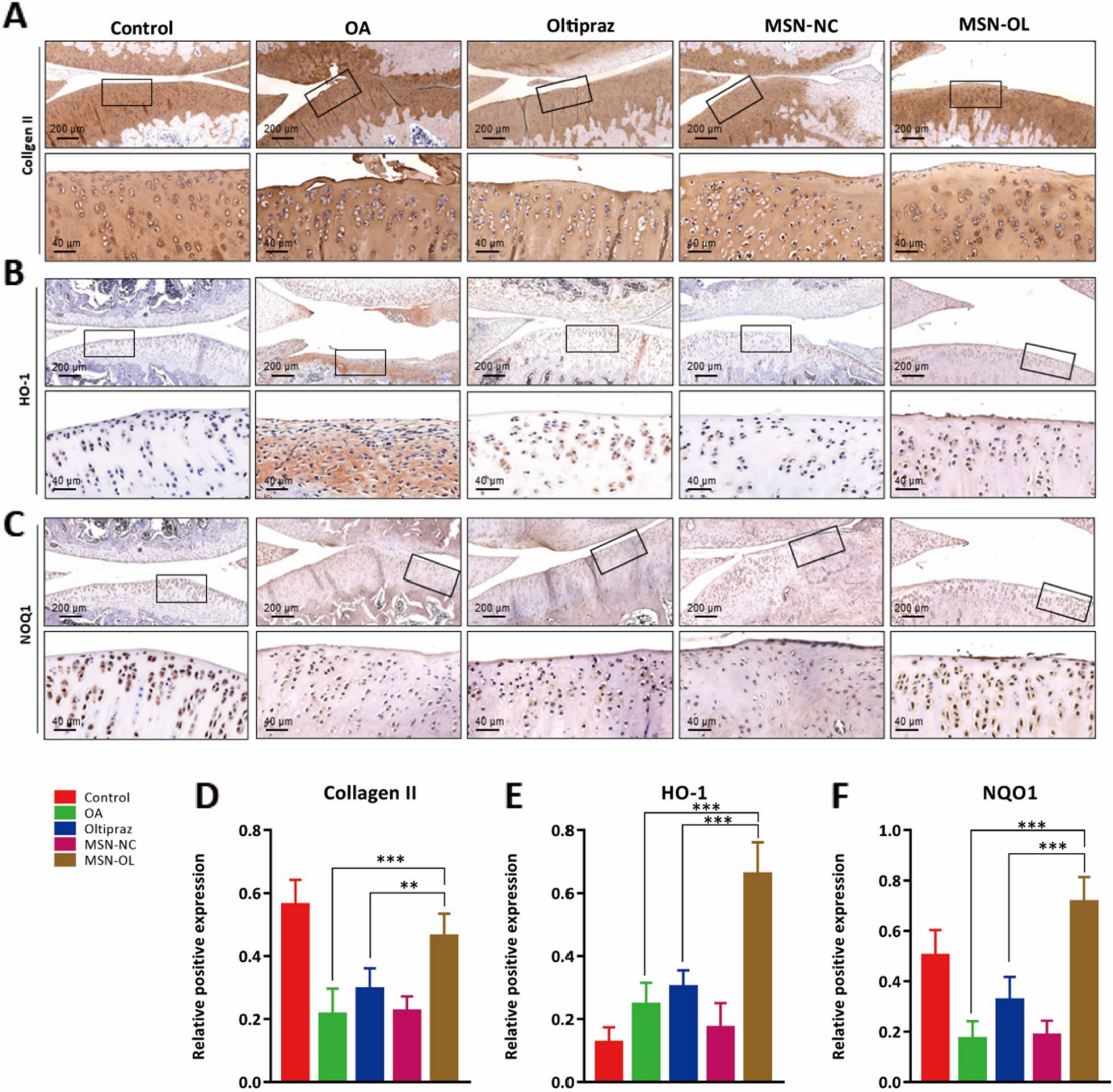
Upregulation of Nrf2/HO-1 signaling pathway after MSN-OL treatment. **A**–**C** Representative images of immunofluorescence staining for collagen II, HO-1, NOQ1. D-F) Quantification of collagen II, HO-1, NOQ1 positive cells in different groups. (** indicated p < 0.01, *** indicated p < 0.001 in comparison with OA or the OL treatment group, respectively)

Furthermore, immunohistochemistry (IHC) analysis showed MSN-OL increased collagen type II expression levels in OA rats compared with OL treatment, suggesting MSN-OL better protected ECM from degradation (Fig. 7A, D). In addition, MSN-OL enhanced the HO-1 (Fig. 7B) and NQO1 (Fig. 7C) protein expression compared with other treatment groups as well. Of note, the HO-1 and NQO1 positive cell ratio in MSN-OL group at 12 weeks were significantly higher than those in OL group (Fig. 7E, F), suggesting that MSN-OL enhanced the activation of Nrf2 signaling pathway and thus promoted HO-1 and NQO1 expression to exert better antioxidative effects in vivo.

## Conclusions

ROS-induced oxidative stress exerts a major pathogenic role in the progression of OA, which causes the apoptosis and dysfunction of chondrocytes and ECM degradation in the cartilage. In this study, we constructed a novel nanocarrier which could take the antioxidants into the chondrocytes and intelligently release the drugs in a high ROS environment. Moreover, the nanocarrier could strongly enhance the biofunctions of antioxidants by activating Nrf2 signaling pathway to exert better antioxidative effects, which provided a paradigm of drug nanocarrier study for OA treatment.

## Supplementary Information

**Additional file 1: Figure S1.** Structural formula of OL. **Figure S2.** Cytotoxicity detection of OL with CCK8 method. **Figure S3.** The intracellular ROS levels of chondrocytes treated by IL-1β. (*** indicated p <0.001 in comparison with the IL-1β treatment group, respectively). **Figure S4.** The results of western blot showed OL activated Nrf2/HO1 signaling pathway. (* indicated p <0.05, ** indicated p <0.01, *** indicated p<0.001, **** indicated p<0.0001). **Figure S5.** The results of type collagen II, aggrecan, MMP9 and MMP13 expression indicated that OL ameliorated IL-1β induced matrix degradation. (* indicated p <0.05, ** indicated p <0.01, *** indicated p <0.001, **** indicated p<0.0001). **Figure S6.** The results of Safranin O/fast green and toluidine blue staining of joint cartilage block showed that OL alleviated IL-1β induced glycosaminoglycan degradation. **Figure S7.** Fluorescence spectra of nanoparticles. **Figure S8.** Zeta potential distribution curve of MSN-OL. **Figure S9.** Dynamic light scattering distribution curve of MSN-OL. **Figure S10.** Cytotoxicity detection of MSN-OL with CCK8 method. **Figure S11.** Flow cytometry was used to determine the uptake of MSN-OL nanoparticles. **Figure S12.** Representative images of H&E staining, Safranin O-fast green staining and Toluidine Blue staining at 4 weeks. **Figure S13.** Representative images of H&E staining, Safranin O-fast green staining and Toluidine Blue staining at 8 weeks.

## Data Availability

The data that support the findings of this study are available from the corresponding author upon reasonable request.
