# Supplementary material for: Cartilage targeting therapy with reactive oxygen species-responsive nanocarrier for osteoarthritis
Source: J Nanobiotechnology. 2022 Sep 19;20:419. doi: 10.1186/s12951-022-01629-w (PMC9484188; doi:10.1186/s12951-022-01629-w)
Supplement: Supplementary file 1 — Additional file 1: Figure S1. Structural formula of OL. Figure S2. Cytotoxicity detection of OL with CCK8 method. Figure S3. The intracellular ROS levels of chondrocytes treated by IL-1β. (*** indicated p <0.001 in comparison with the IL-1β treatment group, respectively). Figure S4. The results of western blot showed OL activated Nrf2/HO1 signaling pathway. (* indicated p <0.05, ** indicated p <0.01, *** indicated p<0.001, **** indicated p<0.0001). Figure S5. The results of type collagen II, aggrecan, MMP9 and MMP13 expression indicated that OL ameliorated IL-1β induced matrix degradation. (* indicated p <0.05, ** indicated p <0.01, *** indicated p <0.001, **** indicated p<0.0001). Figure S6. The results of Safranin O/fast green and toluidine blue staining of joint cartilage block showed that OL alleviated IL-1β induced glycosaminoglycan degradation. Figure S7. Fluorescence spectra of nanoparticles. Figure S8. Zeta potential distribution curve of MSN-OL. Figure S9. Dynamic light scattering distribution curve of MSN-OL. Figure S10. Cytotoxicity detection of MSN-OL with CCK8 method. Figure S11. Flow cytometry was used to determine the uptake of MSN-OL nanoparticles. Figure S12. Representative images of H&E staining, Safranin O-fast green staining and Toluidine Blue staining at 4 weeks. Figure S13. Representative images of H&E staining, Safranin O-fast green staining and Toluidine Blue staining at 8 weeks. [file 12951_2022_1629_MOESM1_ESM.docx]

**Supporting Information**

**Cartilage targeting therapy with reactive oxygen species-responsive nanocarrier for osteoarthritis**

Zengxin Jiang^1,2^, Hao Wang^3^, Zeng Zhang^1,2^, Jianfeng Pan^4,*^, Hengfeng Yuan^1,2,*^

^1^ Department of Orthopaedics, Shanghai Jiaotong University Affiliated Sixth People’s Hospital, Shanghai 200233, China

^2^ Institute of Microsurgery On Extremities, Shanghai Jiao Tong University Affiliated Sixth People's Hospital, Shanghai, 200233, China.

^3^ Department of Orthopaedics, Zhongshan Hospital, Fudan University, Shanghai 200032, China

^4^ Department of Orthopedics, Shanghai Tenth People's Hospital, School of Medicine, Tongji University, Shanghai 200072, China

* Corresponding author

E-mail addresses: pansmith@163.com (J. Pan), yuanhf@shsmu.edu.cn (H. Yuan)


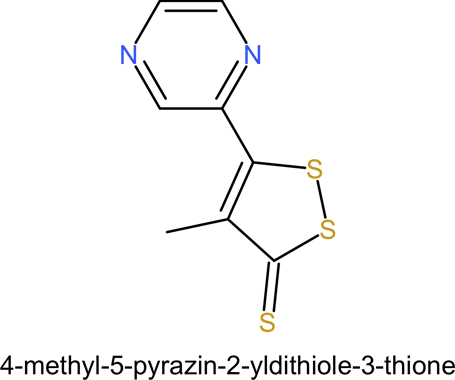


Figure S1. Structural formula of OL.


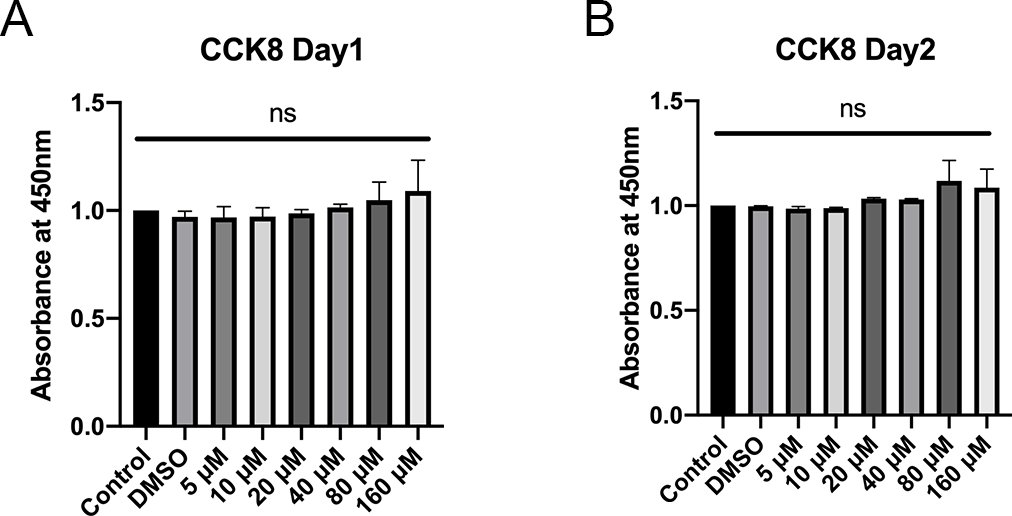


Figure S2. Cytotoxicity detection of OL with CCK8 method.


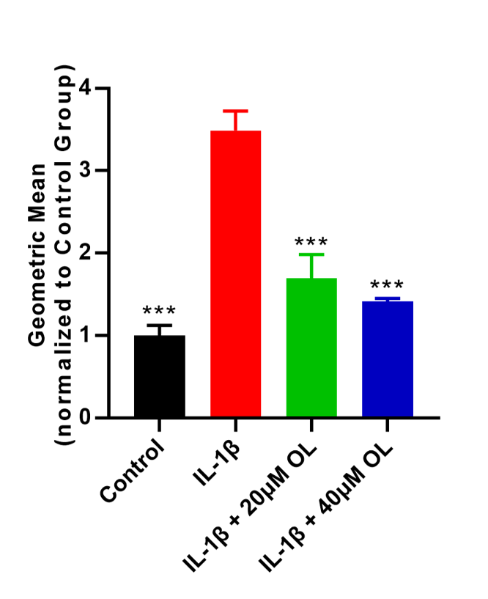


Figure S3. The intracellular ROS levels of chondrocytes treated by IL-1β. (*** indicated p <0.001 in comparison with the IL-1β treatment group, respectively)


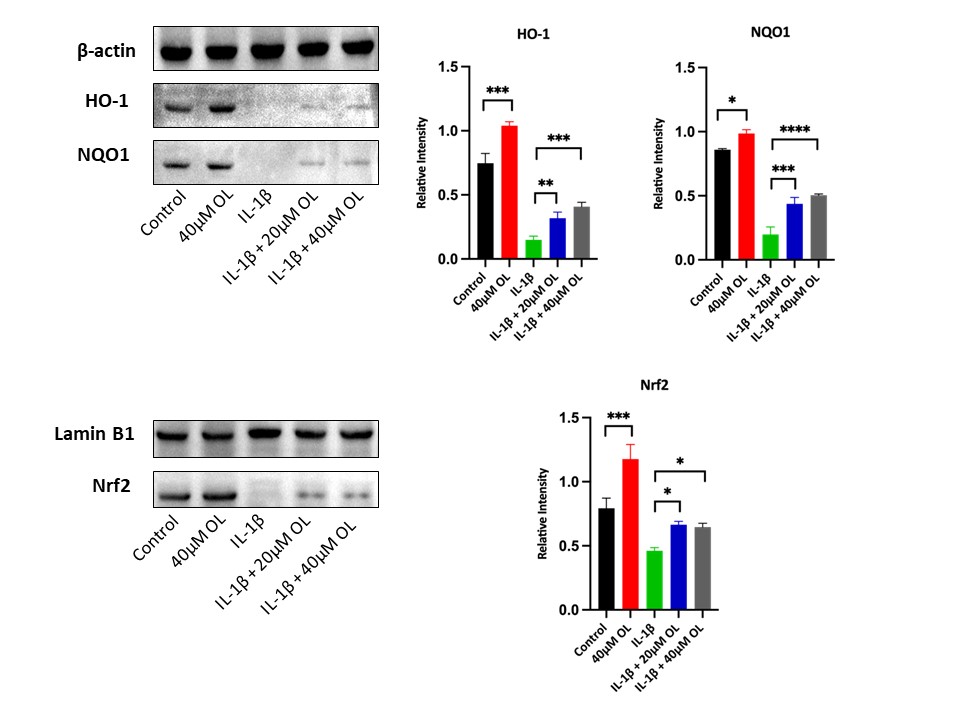


Figure S4. The results of western blot showed OL activated Nrf2/HO1 signaling pathway. (* indicated p <0.05, ** indicated p <0.01, *** indicated p<0.001, **** indicated p<0.0001).


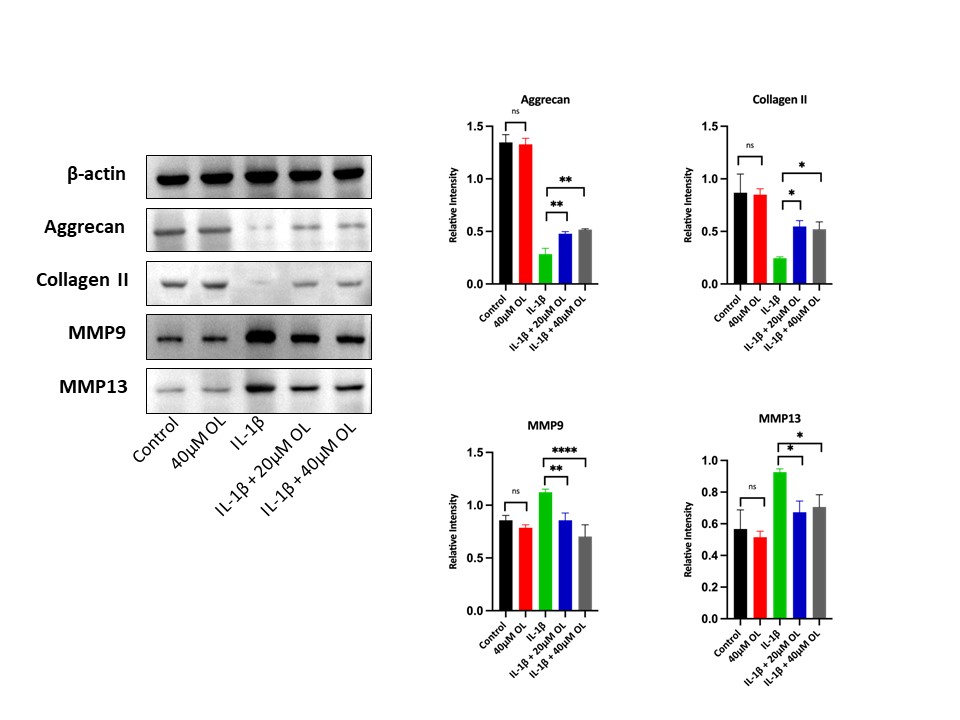


Figure S5. The results of type collagen II, aggrecan, MMP9 and MMP13 expression indicated that OL ameliorated IL-1β induced matrix degradation. (* indicated p <0.05, ** indicated p <0.01, *** indicated p <0.001, **** indicated p<0.0001).


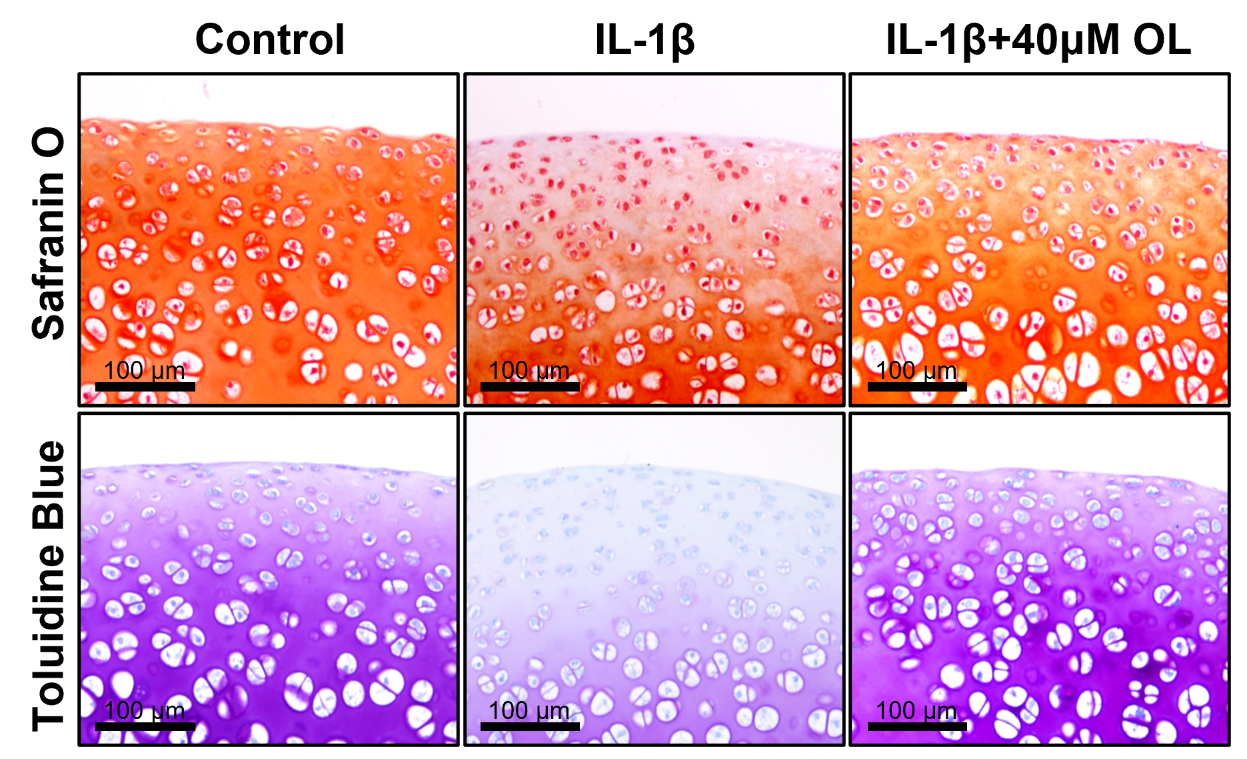


Figure S6. The results of Safranin O/fast green and toluidine blue staining of joint cartilage block showed that OL alleviated IL-1β induced glycosaminoglycan degradation.


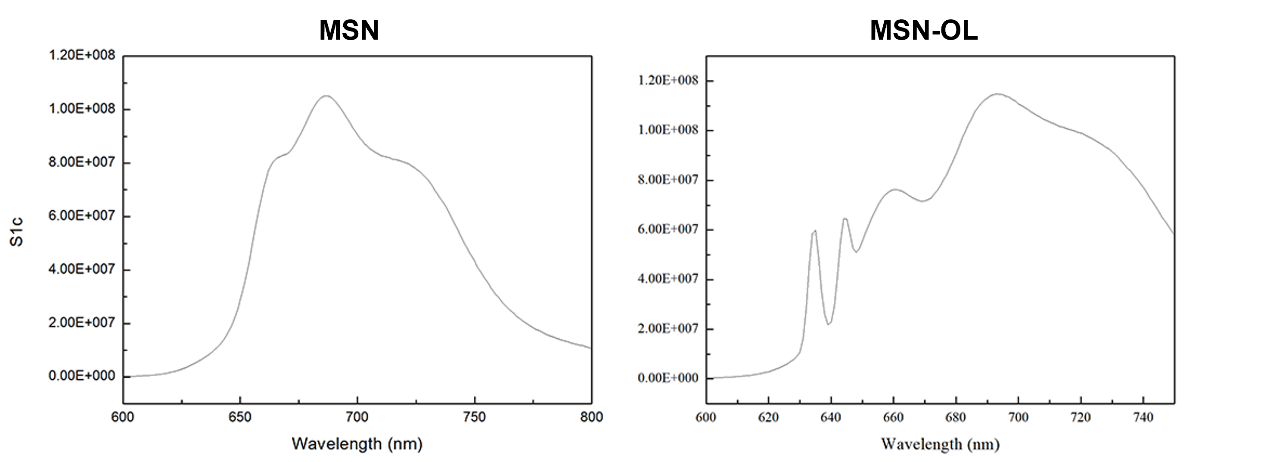


Figure S7. Fluorescence spectra of nanoparticles.


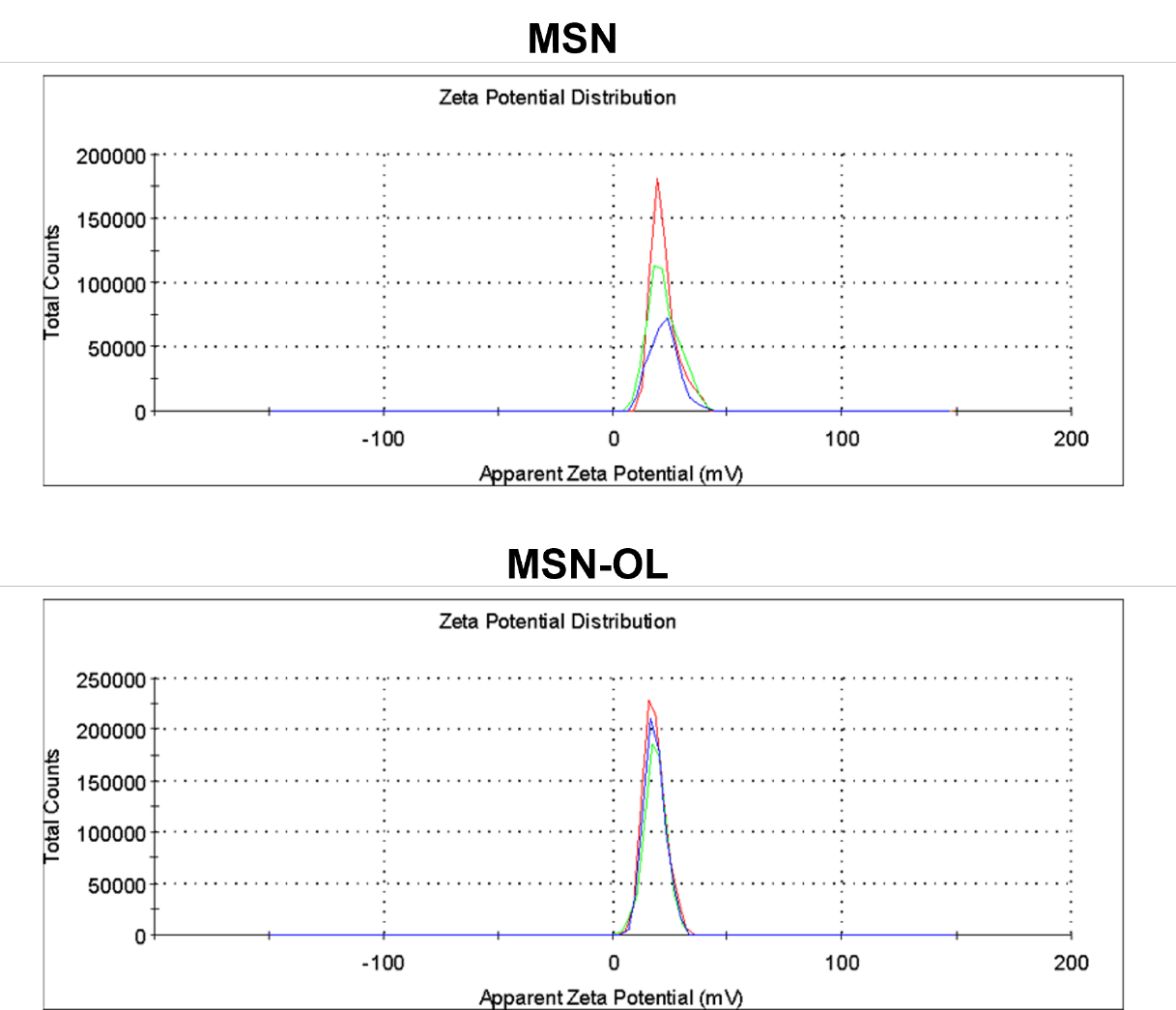


Figure S8. Zeta potential distribution curve of MSN-OL.


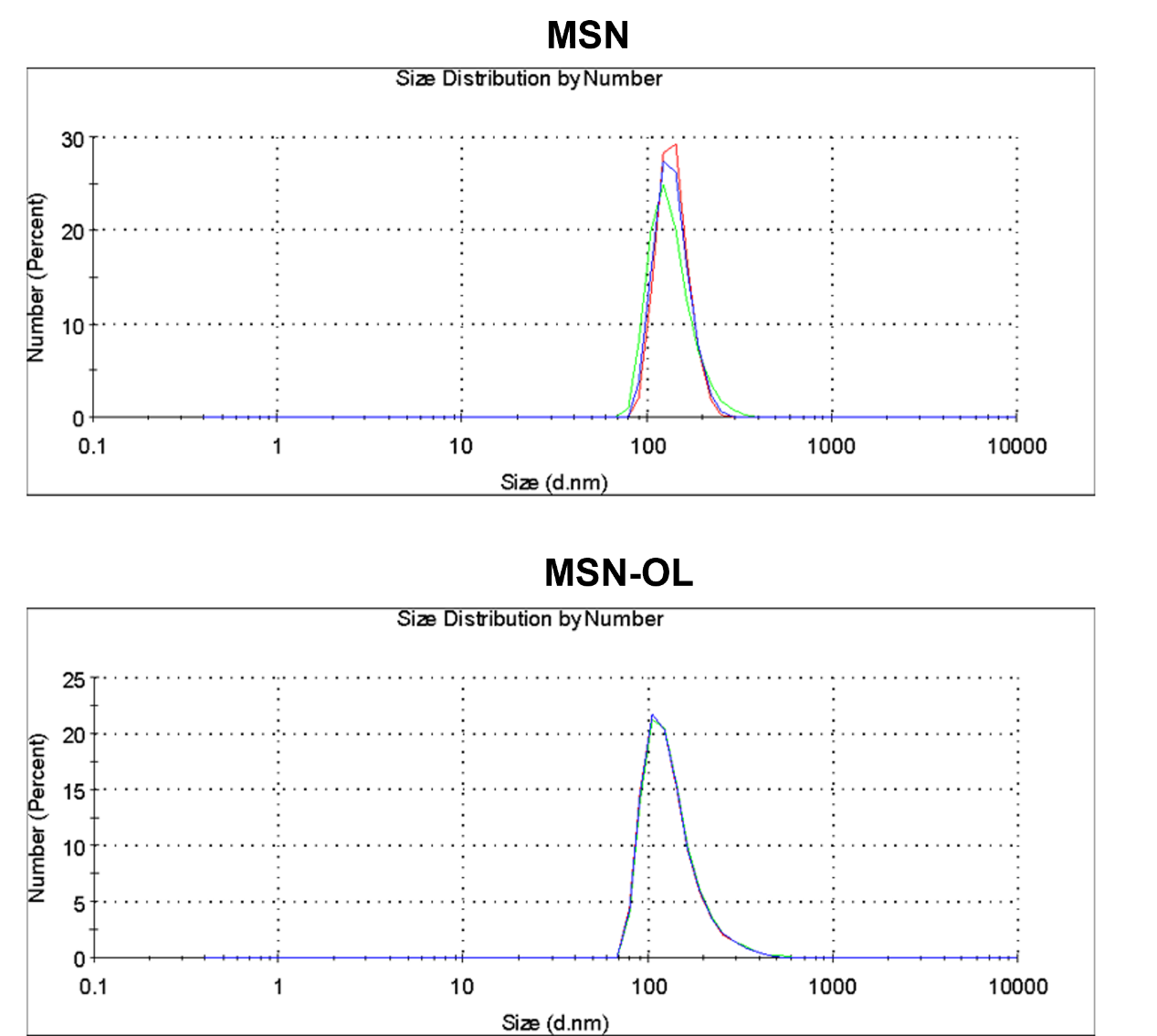


Figure S9. Dynamic light scattering distribution curve of MSN-OL.


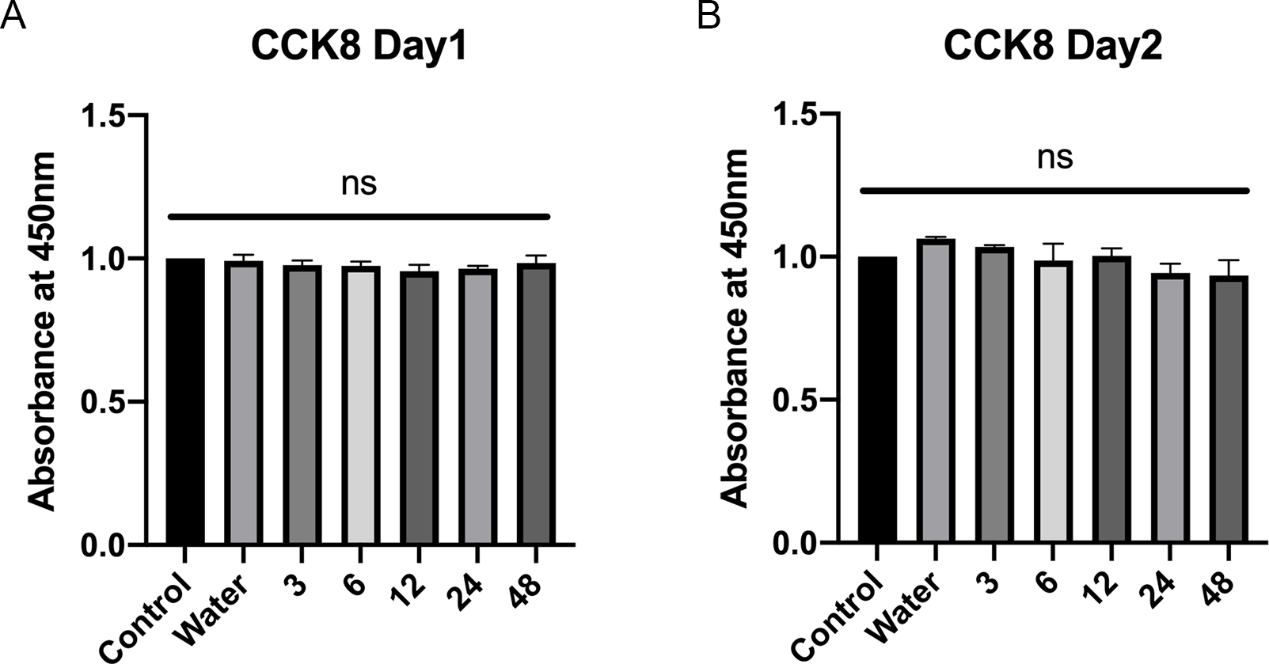


Figure S10. Cytotoxicity detection of MSN-OL with CCK8 method.


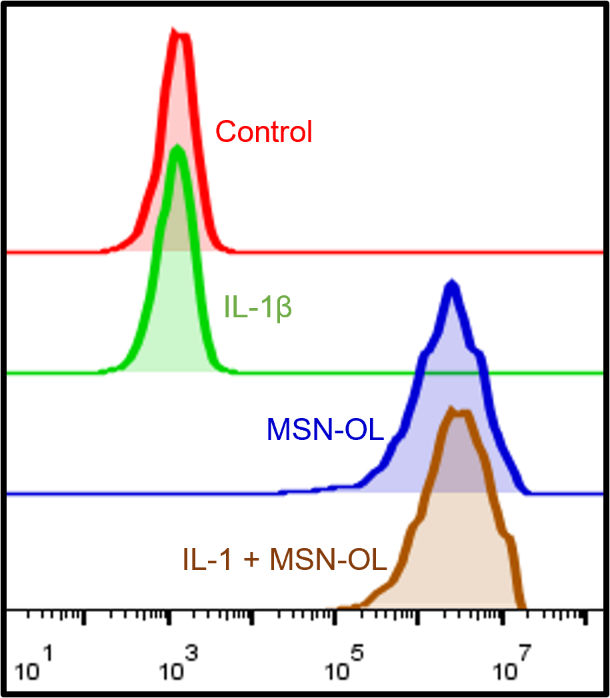


Figure S11. Flow cytometry was used to determine the uptake of MSN-OL nanoparticles


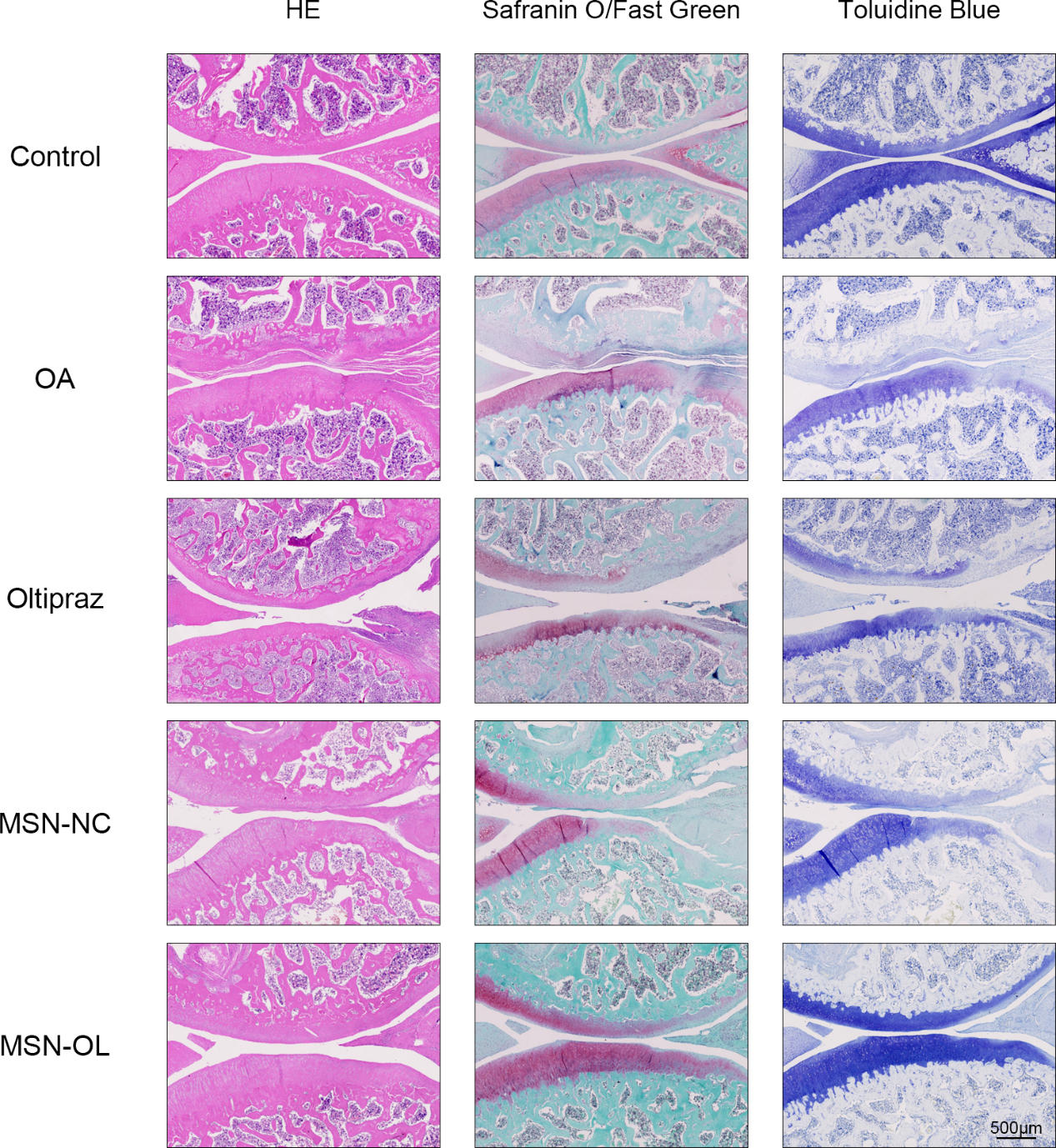


Figure S12. Representative images of H&E staining, Safranin O-fast green staining and Toluidine Blue staining at 4 weeks.


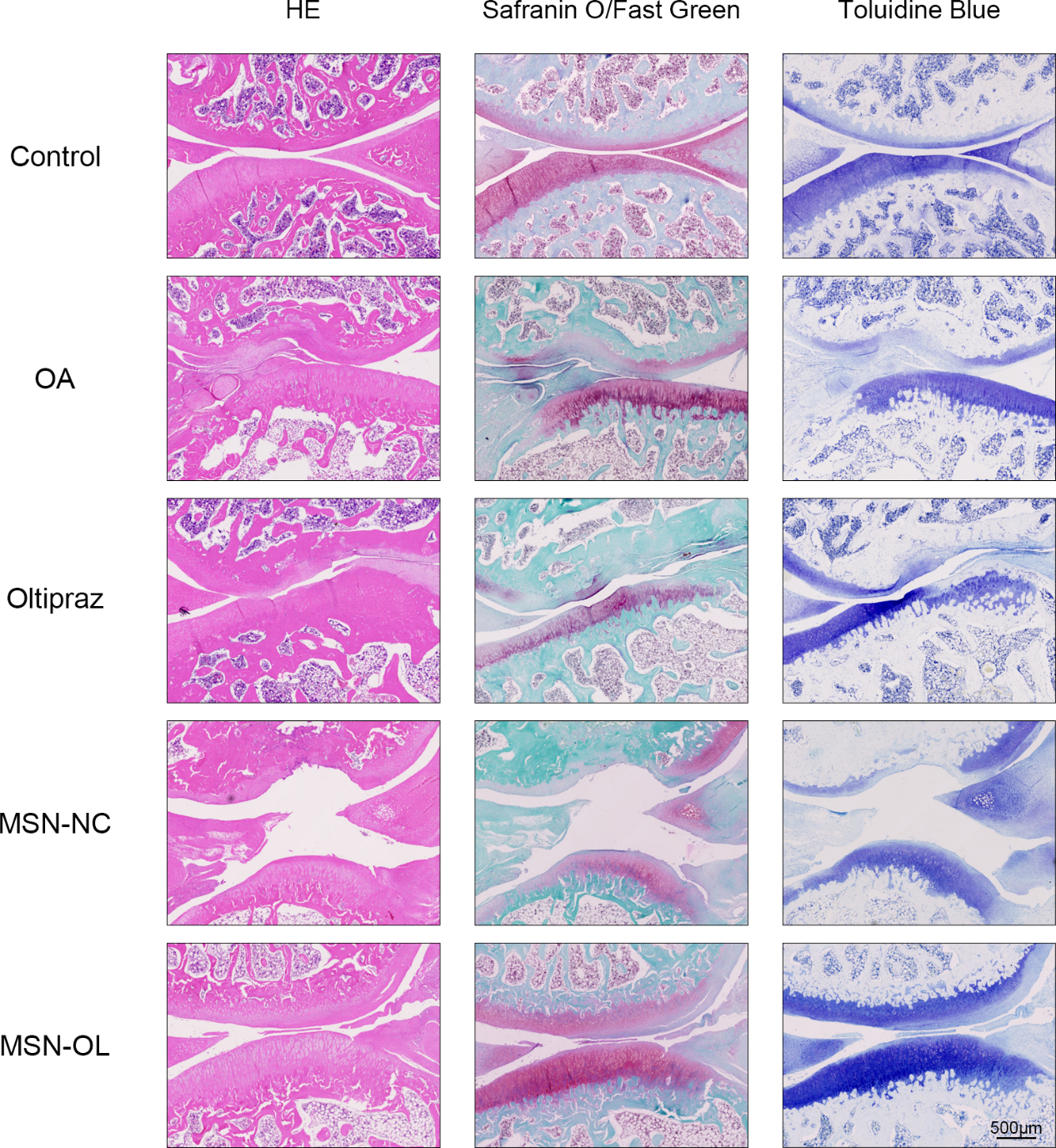


Figure S13. Representative images of H&E staining, Safranin O-fast green staining and Toluidine Blue staining at 8 weeks.
